# Moonlighting in Rickettsiales: Expanding Virulence Landscape

**DOI:** 10.3390/tropicalmed7020032

**Published:** 2022-02-19

**Authors:** Ana Luísa Matos, Pedro Curto, Isaura Simões

**Affiliations:** 1 CNC—Center for Neuroscience and Cell Biology, University of Coimbra, 3004-504 Coimbra, Portugal; anna.matos1999@gmail.com (A.L.M.); pedrocurtobioq@gmail.com (P.C.); 2 IIIUC—Institute of Interdisciplinary Research, University of Coimbra, 3004-504 Coimbra, Portugal

**Keywords:** Rickettsiales, *Anaplasma* spp., *Ehrlichia* spp., *Orientia* spp., *Rickettsia* spp., moonlighting, multitasking, virulence factors, pathogenicity, protein function

## Abstract

The order Rickettsiales includes species that cause a range of human diseases such as human granulocytic anaplasmosis (*Anaplasma phagocytophilum*), human monocytic ehrlichiosis (*Ehrlichia chaffeensis*), scrub typhus (*Orientia tsutsugamushi*), epidemic typhus (*Rickettsia prowazekii*), murine typhus (*R. typhi*), Mediterranean spotted fever (*R. conorii*), or Rocky Mountain spotted fever (*R. rickettsii*). These diseases are gaining a new momentum given their resurgence patterns and geographical expansion due to the overall rise in temperature and other human-induced pressure, thereby remaining a major public health concern. As obligate intracellular bacteria, Rickettsiales are characterized by their small genome sizes due to reductive evolution. Many pathogens employ moonlighting/multitasking proteins as virulence factors to interfere with multiple cellular processes, in different compartments, at different times during infection, augmenting their virulence. The utilization of this multitasking phenomenon by Rickettsiales as a strategy to maximize the use of their reduced protein repertoire is an emerging theme. Here, we provide an overview of the role of various moonlighting proteins in the pathogenicity of these species. Despite the challenges that lie ahead to determine the multiple potential faces of every single protein in Rickettsiales, the available examples anticipate this multifunctionality as an essential and intrinsic feature of these obligates and should be integrated into available moonlighting repositories.

## 1. Introduction

Rickettsiales comprise a diverse and expanding list of vector-borne obligate intra-cellular Gram-negative bacteria that include many animal and human pathogens as well as non-pathogens. Members of Rickettsiales are characterized by small genomes, with sizes ranging between 0.8 and 2.5 Mbp, and the number of hypothetical proteins varying from 88 to 536 (recently reviewed in [1]). Rickettsiales organisms are globally widespread, and their geographical distribution is mostly defined by both vector and natural host constraints. A rise in incidence and resurgence patterns of human diseases caused by these vector-borne pathogens has been increasingly recognized, resulting from the impact of climate and social changes on the distribution and abundance of the arthropod vectors and associated pathogens. For example, human granulocytic anaplasmosis caused by *Anaplasma phagocytophilum* and transmitted by the tick *Ixodes scapularis* is among the three most important vector-borne diseases in the United States (U.S.), with data showing a ~12-fold increase in reported human cases from 2010–2018 [2]. Also, it is increasingly being reported in Asia and several Central/Northern Europe where the distribution of *Anaplasma* and its vector *Ixodes ricinus* is increasing both in latitude and altitude [3,4]. Likewise, the increased incidence of human ehrlichiosis in the U.S. has also been associated with a geographical expansion of the tick *Amblyomma americanum* and *E. chaffeensis* to northern regions [5]. Scrub typhus is currently recognized to have a wider presence beyond the endemic areas of the tropical Asia-Pacific region and northern Australia, with cases emerging in South America, the Middle East, and Africa. It is estimated that approximately 1 million cases of scrub typhus are reported every year, resulting in a significant and increasing burden in endemic areas [6,7]. Interestingly, although it is known that chigger trombiculid mites (*Leptotrombidium* spp.) are the vector for *Orientia tsutsugamushi*, the nature of the vector(s) of other emerging *Orientia* species in the Western Hemisphere is still not clear [6]. Among rickettsial diseases, there is a growing concern about the globally increasing incidence of Spotted fever group rickettsioses (SFR); not only the most severe forms of these diseases but, particularly, milder forms caused by new species of rickettsiae [8,9]. For example, the incidence of SFR in the U.S. has significantly increased over the last two decades, from 1.7 to 19.2 cases per million persons from 2000 to 2017 [10,11]. Rocky Mountain spotted fever (RMSF), caused by *R. rickettsii* through a bite of *Dermacentor variabilis*, *D. andersoni*, or the brown dog tick (*Rhipicephalus sanguineus*), is still the most severe and fatal of the SFR. Recent outbreaks of RMSF in Mexico (2004–2016) resulted in case fatality rates of ~29–30% (higher in children <10), representing an expanding public health problem [12]. Mediterranean spotted fever, caused by *R. conorii* (and transmitted by *R. sanguineus*) is endemic to the Mediterranean basin and considered the most prevalent rickettsial disease in Europe [13]. There is also a growing concern in Europe about the increasing incidence of milder rickettsioses, such as tick (*Dermacentor*)-borne lymphadenopathy/Dermacentor-borne necrotic erythema and lymphadenopathy (TIBOLA/DEBONEL) syndrome [14], and the epidemiological importance of SFRs in Africa is increasingly recognized [15]. Importantly, rickettsial diseases transmitted by fleas like murine typhus and flea-borne spotted fever (caused by *R. typhi* and *R. felis*, respectively) occur worldwide, and emergence patterns for both are evident in many regions of the globe [16,17]. Other *R. felis*-like organisms, like *R. asembonensis*, follow identical geographical patterns as those of *R. felis* and have been associated with other arthropods, raising the interest in understanding in detail their potential as a pathogen [18].

The drastic increase in the identification of new Rickettsiales species whose pathogenicity to humans is still uncertain, increased probabilities of Rickettsiales-carrying arthropods to encounter human hosts, the high mortality rates of some of these diseases if left untreated, the fact that these organisms are not susceptible to many classes of antibiotics, and the absence of approved vaccines bolsters the need to continue understanding the pathogenesis of these obligate pathogens and identifying new therapeutic targets for these diseases [9,19].

Moonlighting proteins represent a vast group of virulence factors in many pathogenic bacteria. Given the reduced genomes of the organisms belonging to Rickettsiales, understanding the importance of this multitasking phenomenon to expand their virulence landscape is of paramount relevance. Herein, we contextualize the concept with a brief introduction on bacterial moonlighting/multitasking proteins, and then provide an overview of the current knowledge on moonlighting among the different members of Rickettsiales, discussing the relevance of this emerging theme and how it will impact our fundamental understanding of protein function.

## 2. Bacterial Moonlight Proteins

Moonlight proteins comprise a subset of proteins that present more than one physiologically relevant function that is not due to gene fusion, multiple RNA splice variants, or pleiotropic effects [20]. Another characteristic of these proteins is that the functions exerted are independent of each other, meaning that the inactivation of one function must not affect the others. According to MoonProt 3.0 [21] (MoonProt 3.0. Available online http://moonlightingproteins.org (accessed on 5 February 2022) and MultitaskProtDB-II [22] (MultitaskProtDB-II. Available online: http://wallace.uab.es/multitaskII accessed on 5 February 2022), two repositories where these activities are being compiled, there are over 500 annotated moonlight/multitasking proteins which are extraordinarily diverse and involved in a large array of biological functions. Since many of the proteins have evolved to exert various functions, there is not a shared sequence or structural feature that can be used to foresee the moonlighting activity, being mainly discovered by serendipity, proteomics, and yeast two-hybrid assays [23]. Additionally, if a protein is homologous to a moonlighting/multitasking protein, it does not imply that it presents the same functions since it can exert one, both, or none of the moonlighting protein functions [24]. Due to this, it is difficult to identify and predict if a protein presents moonlight activity. However, bioinformatics approaches have been in development to aid in this process [25,26,27]. 

Moonlight proteins are present in several organisms, including animals, plants, yeasts, bacteria, and viruses [24,28]. These proteins have been gaining attention by playing important roles in autoimmune diseases, diabetes, and cancer [29]. In bacteria, moonlighting proteins are present in both pathogenic and commensal bacteria, and many of these proteins are highly conserved in these organisms [30]. Importantly, 25% of moonlight proteins are classified as virulence factors and may contribute to bacterial pathogenesis and virulence [31,32]. Virulence factors are expressed and secreted by the pathogen, allowing it to colonize the host, establish infection, and evade the immune system, and can be classified into different groups, depending on their function [33]. Cytosolic factors allow bacteria to adapt to the host environment by undergoing quick adaptative metabolic, physiological, and morphological shifts. Membrane-associated factors facilitate bacteria adhesion and invasion of host cells, and secretory factors allow them to evade the host’s innate and adaptive immune system as well as cause tissue damage [33]. Due to this, moonlight proteins are good targets to treat bacteria-caused diseases because they can be involved in many steps of infection. Major groups of bacterial moonlight proteins implicated in virulence include metabolic enzymes of the glycolytic and other metabolic pathways, molecular chaperones, and protein-folding catalysts [34]. Among these, most moonlighting virulence factors mediate adhesion and modulation of leukocyte activity.

Proteins with moonlight activity often exert their canonical function in a different cell compartment of their moonlight function. One example is the glycolytic enzyme Glyceraldehyde 3-phosphate dehydrogenase (GAPDH), the first identified bacterial moonlight protein [35]. GAPDH catalyzes the conversion of D-glyceraldehyde 3-phosphate to 3-phosphoD-glyceroyl phosphate in glycolysis when present inside the cell, and on the cell surface of group A streptococci can present adhesive functions in vitro [36]. Besides presenting more than one function in group A streptococci, GAPDH moonlighting activity is also present in other species. In *Bacillus anthracis*, *Lactobacillus crispatus*, *Staphylococcus aureus*, and *Staphylococcus pneumoniae* GAPDH also binds to plasmin. Furthermore, GAPDH can bind fibronectin, an extracellular matrix component (ECM), and bind complement protein C5a, mediating immune evasion, among other described functions [35]. 

Bacterial adherence factors are cell surface proteins that form and maintain physical interactions with host cells and tissues. This feature is crucial in pathogenic bacteria because it promotes infection but is also important in commensal bacteria to maintain a symbiotic relationship with the host [32]. Enolase is a well-characterized moonlight protein that converts 2-phosphoglycerate to phosphoenolpyruvate in glycolysis and can exert other functions on the surface of pathogens [32]. Enolase can bind to the host’s ECM or airway mucins and can bind to the coagulation cascade protease plasminogen in *Borrelia burgdorferi*, *Candida albicans*, *Streptococcus pneumoniae*, among others, contributing to bacterial virulence [37]. 

Moonlight cytosolic proteins can be secreted as signaling molecules, regulating various cell types in an organism or modulating host responses in the case of a pathogen. In the cytosol, chaperones are proteins that bind and assist the folding or unfolding of macromolecular structures in order to prevent misfolding and promote reassembly and correct assembly of protein complexes [32]. In mammals, heat shock protein 60 (Hsp60), GroEL, is a chaperone that mediates mitochondrial protein import in the cell [32]. However, it has been demonstrated that bacterial Hsp60/GroEL can mediate adhesion in several species, including *Clostridium difficile*, *Helicobacter pylori*, and *Chlamydia pneumoniae*, contributing to their virulence [32]. Elongation factor (EF)-tu is another example. It is a cytoplasmic protein important in protein synthesis that transports and catalyzes the binding of aminoacyl-tRNA to the ribosome. However, besides its canonical function, EF-tu can act also as an adhesin at the surface of several pathogens such as *Mycobacterium pneumoniae* and *Pseudomonas aeruginosa* by binding fibronectin and plasminogen, respectively [38,39]. 

Among these multitasking proteins are also proteolytic enzymes mainly located in the membrane or secreted and recognized as virulence factors in various pathogenic bacteria [40]. These molecules cleave specific peptide bonds of target proteins, resulting in activation, inactivation, generation of new proteins, and even changing protein function, being important in colonization and evasion of the host immune defenses, acquisition of nutrients for growth and proliferation, and facilitation of dissemination or tissue damage during infection [41,42]. For example, streptococcal C5a peptidase is a surface serine protease that cleaves and inactivates complement protein C5a. Furthermore, this protease can mediate fibronectin binding, acting as an adhesin. This moonlight function is important for streptococci virulence, helping in the attachment and invasion of eukaryotic cells [43]. Additionally, *Escherichia coli* presents the protease OmpT, which acts as an adhesin to human cells [44,45]. 

## 3. Intracellular Bacterial Moonlight Proteins

Moonlight activity is not exclusive of extracellular bacteria, being present in various intracellular pathogens such as *Listeria monocytogenes*, *Shigella flexneri*, *Legionella pneumophila*, and *Mycobacterium tuberculosis*. These organisms are responsible for causing diseases of global importance, as is the case of Legionnaire’s disease or tuberculosis, resulting in significant morbidity and mortality [46]. Intracellular bacteria need the host cells to efficiently replicate and proliferate during infection, escaping the hosts’ immune system [47]. Most species enter the host through disruptions in the mucosae and the skin; however, others can enter directly into the bloodstream via bites of vectors such as mosquitoes and ticks [48]. Furthermore, intracellular pathogens can multiply into several cell types, such as epithelial cells, fibroblasts, monocytes/macrophages, and dendritic cells [49].

Intracellular bacteria can form actin tails inside the host cells in order to spread from cell to cell. This structure is important in bacterial virulence and helps to spread the infection. Actin-based motility (ABM) requires actin polymerization and is mediated by the expression of bacterial factors that hijack host cell actin nucleation machinery or exhibit intrinsic actin nucleation properties [50]. Besides ABM factor’s canonical function, they can also display adhesion functions such as bacterial aggregation, biofilm formation, and host cell adhesion/invasion. Some ABM factors that exert this moonlight function are *L. monocytogenes* ActA and *S. flexneri* IcsA [50] (among others, as shown later). 

As previously described, Hsp60 chaperone mediates adhesion in several species [32]. In *S. typhimurium*, Hsp60 also mediates bacterium aggregation to intestinal mucus [51]. Moreover, it was also observed that Hsp65 inhibition decreases bacterial binding in the intestine, suggesting its role in this early step of infection [51]. *Listeria* adhesion protein (LAP) is an alcohol acetaldehyde dehydrogenase that acts as an adhesin in *L. monocytogenes*, it also being a moonlighting protein [52]. Hsp60 from *L. monocytogenes* interacts with LAP and promotes bacterial adhesion in enterocyte-like cells [53]. Furthermore, *L. pneumophila* Hsp60’s presence on the cells’ surface can also mediate invasion into non-phagocytic cells [54]. Bacterial Hsp70, DnaK, is the most conserved and ubiquitous heat shock protein [55]. Hsp70 can bind to plasminogen (a serine protease involved in the coagulation cascade) and cause its subsequent conversion to plasmin, the active form of the protease. This promotes the host ECM and basement membrane degradation, aiding host tissue invasion [34,37]. Furthermore, *M. tuberculosis* DnaK can bind to the cluster of differentiation 40 (CD40), contributing to the synthesis of monocyte chemokines and the maturation of dendritic cells [56].

GAPDH also presents moonlight activity in intracellular bacteria. Besides its canonical function in glycolysis, GAPDH can be present on the surface of *L. monocytogenes* and *S. aureus* and bind plasmin, being important in the invasion and proliferation of these pathogens [35]. Moreover, enolase can bind ECM components plasminogen and laminin in *S. aureus*, contributing to the invasion of host tissues [37]. Other metabolic enzymes such as malate synthase from *M. tuberculosis* can act as an adhesin by binding fibronectin and laminin [57]. Moreover, *M. tuberculosis* glutamine synthetase can bind fibronectin and plasminogen [58]. Bacterial autolysins are peptidoglycan hydrolases that break down peptidoglycan components of cells, being important in cell division, separation, and cell wall turnover [59]. Aaa autolysin present in *S. aureus* presents adhesive properties by binding fibronectin [60]. Furthermore, Ami autolysin from *L. monocytogenes* can also mediate adherence to eukaryotic cells [61].

## 4. Moonlighting in Rickettsiales

The order Rickettsiales comprises a diverse group of Gram-negative alphaproteobacteria with an obligatory intracellular lifestyle and a mandatory transmission cycle that includes arthropods as hosts, reservoirs, and vectors [62]. The order is divided into two families (*Anaplasmataceae* and *Rickettsiaceae*), seven major genera (*Anaplasma*, *Ehrlichia*, *Wolbachia*, *Neorickettsia*, *Rickettsia*, *Orientia*, and SAR11), and comprises at least 87 species that exhibit high diversity in cell structure, vector preference, host cell type preference, pathogenicity and infection cycle [1].

Organisms of the order Rickettsiales have been found in all continents and are associated with diverse habitats in which arthropod species such as fleas, lice, ticks, and mites are present [63,64,65]. Rickettsiales organisms can be transmitted to various mammalian hosts, including humans and animals (e.g., rodents, cattle), through the saliva or feces of feeding arthropods [64]. Rickettsial pathogens are inoculated into the blood or skin during the blood meal and are disseminated through the body via the blood and/or lymphatic system [64]. Once inside the mammalian host, the tropism to mammalian cells can vary between species infecting erythrocytes (*Anaplasma marginale*), endothelial cells (*Rickettsia* spp., *Orientia tsutsugamushi*), dendritic cells (*Orientia tsutsugamushi*), neutrophils (*Anaplasma phagocytophilum*), or macrophages (*Orientia tsutsugamushi*, *Ehrlichia chaffeensis*, and some *Rickettsia* spp.) [1].

The order Rickettsiales includes species that can be non-pathogenic, animal pathogens, and human pathogens [1]. The clinical spectrum of rickettsial disease in humans varies widely, ranging from mild illness to rapidly fatal disease [66,67]. The extensive range of human diseases caused by Rickettsiales organisms includes human granulocytic anaplasmosis (*Anaplasma phogocytophilum*), human monocytic ehrlichiosis (*Ehrlichia caffeensis*), human granulocytic ehrlichiosis (*Ehrlichia ewingii*), scrub typhus (*Orientia tsutsugamushi*), epidemic typhus/Brill-Zinsser disease (*Rickettsia prowazekii*), rickettsialpox (*Rickettsia akari*), Mediterranean spotted fever (*Rickettsia conorii*), and Rocky Mountain spotted fever (*Rickettsia rickettsii*), among many others [1,66].

Historically, rickettsial agents such as *R. prowazekii* have been important causes of human morbidity and mortality, being associated with several million deaths in the Union of Soviet Socialist Republics (USSR) [68]. Despite significant advances in science/modern medicine, diseases caused by Rickettsiales species remain a major public health concern. For example, scrub typhus threatens one billion people and causes nearly a million cases per year in the Asia-Pacific area [67,69]. A systematic review on the burden of scrub typhus in India revealed that scrub typhus accounts for at least 25.3% among individuals with acute undifferentiated febrile illness [70]. 

Economic globalization, changes in land use and urbanization, increase in travel, and global warming have all been postulated to raise the distribution and incidence of diseases caused by Rickettsiales [71,72]. Indeed, these diseases are gaining global momentum because of their resurgence patterns, being reported not only in previously endemic regions but also new regions [73,74]. As already mentioned, climate and environmental changes have already contributed to expanding the range of several tick species into higher latitudes in North America, thus resulting in the emergence of anaplasmosis and other tick-borne diseases [75,76]. Global warming has also been associated with the increased occurrence of scrub typhus, with 1 °C rise in temperature causing an increase of 15% in monthly cases [77].

**Figure 1:**
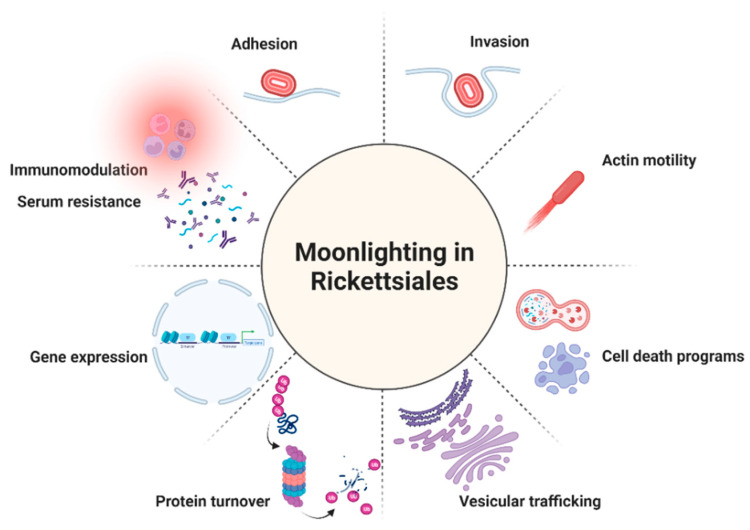
The multiple roles of the reported moonlighting/multitasking proteins in Rickettsiales. Created with BioRender.com.

As summarized in the previous sections, moonlighting/multitasking proteins contribute significantly to the population of virulence factors employed by bacteria to aid colonization and induce disease. Strikingly, by searching MoonProt 3.0 or MultitaskProtDB-II, we could only find one protein from Rickettsiales organisms annotated in the second database, the parvulin-like PPIase from *R. prowazekii*. However, and as we will try to demonstrate in the following subsections, “multitaskers” represent a growing class of proteins among these obligate bacteria, highlighting the relevance of this phenomenon to expand their capacity to interfere with multiple host cell processes (Figure 1). 

### 4.1. Anaplasma spp.

Enolase is suggested to exert multiple functions in *Anaplasma phagocytophilum*. This species causes human granulocytic anaplasmosis, an emerging zoonosis in the U.S., Asia, and Europe. Interestingly, besides transmission via bites of infected vectors, transmission has been reported through blood transfusion and contact with infected mammal blood [78]. Through ELISA assays, Gao and colleagues demonstrated that recombinant enolase from *A. phagocytophilum* can bind and activate plasminogen, being crucial to help pathogen infection [79]. Enolase has been identified as an important candidate antigen for vaccines, being critical to evaluate if this protein can be a good target to control anaplasmosis infection [79]. Nonetheless, further experiments need to be conducted to validate the role of enolase in *A. phagocytophilum* pathogenesis. 

Acquisition of host nutrients by intracellular bacteria is crucial for their pathogenesis and development of the disease. Rab GTPases control intracellular vesicular trafficking by recruiting effector molecules and aid in the infection step by redirecting vesicular traffic to *A. phagocytophilum* occupied vacuole (ApV) [80]. Rab10 regulates ER structure and dynamics and also localizes to the ApV [80,81]. Truchan and colleagues have demonstrated that Rab10 is important to parasitize exocytic traffic from the trans-Golgi network (TGN) [80]. TGN vesicles containing Rab10 localized in the ApV lumen are a source of sphingolipid-rich contents that are acquired by the bacteria. Interestingly, Rab10 binds to uridine monophosphate kinase (UMPK), which was demonstrated to be present on the surface of *A. phagocytophilum*. This protein’s annotated function as catalyzing the conversion of uridine monophosphate to uridine diphosphate in the cytoplasm suggests that UMPK might also be a moonlighting protein [80] (as already demonstrated for *E. coli* UMPK). However, further studies are needed to validate this in *A. phagocytophilum*. 

Bacterial secretion systems are a subset of proteins complexes on the cell membrane that are important in pathogenic bacteria to secrete virulence factors that help in the invasion of host cells. Ankyrin repeat domain-containing protein A (AnkA) is a T4SS effector protein that is found in the nuclei of granulocytes. AnkA binds a broad range of DNA and protein in the nucleus of infected cells and might contribute to modulating neutrophils to facilitate their survival and replication [82]. Garcia-Garcia and colleagues demonstrated that AnkA interacts with CYBB promoter, regulating CYBB locus and causing downregulation of expression of this defense gene as well as other host defense genes. Moreover, AnkA binds DNA regions that display high AT contents and recruits histone deacetylase 1 (HDAC1) to repress gene expression [83,84]. Tyrosine phosphorylation of proteins is crucial in signal transduction in eukaryotic cells. Pathogens can interfere with this pathway by presenting virulence factors that undergo tyrosine phosphorylation, enabling destabilization of host cell functions [85]. AnkA is an example of that as it undergoes tyrosine phosphorylation by Src kinase early during infection. Furthermore, phosphorylated AnkA interacts with neutrophil phosphatase Src Homology Protein (SHP)-1, interfering with host cell signaling and potentially facilitating the survival of *A. phagocytophilum* [85]. AnkA also binds the adaptor protein Abl-interactor 1 (Abi-1) and is phosphorylated by tyrosine kinase Abl-1 [86]. Lin and colleagues have demonstrated that this interaction is important in infection since the inhibition of Abl-1 reduces phosphorylated AnkA and inhibits *A. phagocytophilum* infection. On the other hand, the inhibition of Src kinase did not affect bacterial infection [86]. Finally, AnkA displays another function, interacting with actin and actin-regulating proteins α-actinin 4 and gelsolin. This suggests that AnkA also acts as a protein scaffold that modulates actin dynamics in bacterial growth, development, and release [87]. 

*Anaplasma* translocated factor 1 (Ats-1) is another T4SS effector protein secreted by *A. phagocytophilum* that has moonlight activity. This effector is imported into the mitochondria of infected cells where the mitochondria-targeting presequence is cleaved, forming its mature form [88]. Apoptosis is an important defense mechanism in cells to prevent infection in the host. Pathogens have evolved several ways to modulate cell death pathways, contributing to their pathogenesis [89]. Mature Ats-1 inhibits apoptosis in mammalian cells by inhibiting cytochrome c release from mitochondria and PARP cleavage, and also inhibits Bax-induced apoptosis in yeast cells [88]. Autophagy is a key cellular process that degrades unnecessary or dysfunctional components, including intracellular pathogens, being considered a crucial immune response mechanism [90]. Beclin 1 is a subunit of the phosphatidylinositol 3-kinase catalytic subunit type 3 (PI3KC3) autophagy initiation complex. When this complex is activated, it leads to the formation of the earliest stage of autophagosome [91]. Ats-1 binds Beclin 1 and Atg14L, an ER protein involved in autophagosome nucleation, inducing autophagosome formation [91]. The autophagosomes fuse with *Anaplasma* inclusions, delivering autophagic cargo that can be used as nutrients by the pathogen. The ectopic expression of Ats-1 in infected cells resulted in *Anaplasma* inclusions targeting and enhancing infection by promoting bacterial growth [90]. Thus, Ats-1 can also act to recycle host nutrients and membranes to facilitate bacterial multiplication, contributing to its pathogenesis.

The chaperones GroEL and HSP70 also present multiple functions in *A. phagocytophilum*. Protein levels of these chaperones are increased in high-percentage *A. phagocytophilum*-infected tick cells and salivary glands. Villar and colleagues demonstrated that HSP70 and GroEL are present on the surface of this pathogen and bind to *I. scapularis* tick cells [92]. Through immunoprecipitation and pull-down assays, the interaction between HSP70, GroEL and major surface protein 4 (MSP4) was observed, suggesting the formation of a complex on the membrane of *A. phagocytophilum* that aids the interaction with tick cells. Furthermore, *E. coli* expressing recombinant GroEL and HSP70 with truncated peptide-binding domains did not bind to tick cells, supporting the role of these proteins in pathogen-vector interactions [92].

### 4.2. Ehrlichia chaffeensis

*Ehrlichia chaffeensis* tandem repeat protein (TRP) effectors secreted by the type 1 secretion system (T1SS) and their diverse and continually expanding roles in infection have been extensively reviewed recently [93]. Among TRPs, TRP120 effector is considered a model moonlighting protein [93,94]. The most well-documented functions of this effector comprise ligand mimic, nucleomodulin, and E3 ubiquitin ligase activity, although additional roles have been proposed [93]. TRP120 interacts with Wnt and Notch receptors to activate both pathways, regulating apoptosis and autophagy and promoting bacterial survival. Furthermore, TRP120 stability and interaction with Wnt FZD receptors stimulate bacterial internalization and the infection of host cells [95,96]. This effector also modulates the expression of several genes and, therefore, regulates multiple processes, including cytoskeleton organization and apoptosis [93]. TRP120 interacts with GC-rich regions of DNA, and its acidic nature facilitates interaction with negatively charged DNA [97]. TRP120 also presents E3 ubiquitin ligase activity, which allows the regulation of signaling pathways (including Notch) by targeting host proteins to degradation [93]. 

The 200 kDa ankyrin repeat protein (Ank200) is also an effector secreted by the type 1 secretion system that presents various functions. Ank200 is present in the nucleus during infection and acts as a nucleomodulin [94]. Moreover, Ank200 targets transcriptional factors, genes of the epigenetic machinery and components of signaling pathways including the Jak-Stat pathway to reprogram the host cell and cause immune subversion. This protein also targets genes involved in vesicular trafficking and cytoskeleton proteins and might contribute to the delivery of nutrients to ehrlichial inclusions and consequently increase its replication [94].

*E. chaffeensis* secretes ehrlichial translocated factor 1 (Etf-1), a T4SS effector that is the ortholog of *Anaplasma* Ats-1 [87]. As described for Ats-1, Etf-1 is present in the mitochondria and inhibits apoptosis in mammalian cells and yeast [98]. Antioxidant mitochondrial manganese superoxide dismutase (MnSod) increases upon infection with this pathogen. On the other hand, reactive oxygen species (ROS) significantly decrease in both *E. chaffeensis*-infected cells and Etf-1-infected cells, preventing cellular damage and apoptosis. Inhibition of this effector results in a significant decrease of bacterial infection, being essential for the pathogenesis of *E. chaffeensis* [98]. Etf-1 also modulates autophagy. GTPase RAB5 regulates early endosome maturation to late endosomes and consequently the fusion of light chain 3 (LC3)-positive autophagosomes with late endosomes [99]. Etf-1 recruits GTP-bound RAB5 and binds autophagic proteins VPS34 and Beclin1, activating class III phosphatidylinositol 3-kinase (PtdIns3K) [100]. These RAB5 endosomes traffic and fuse with ehrlichial inclusions, contributing to autophagy enhancement, avoiding autolysosomal killing, and promote bacterial replication by providing nutrients for its growth [100].

### 4.3. Orientia tsutsugamushi

*O. tsutsugamushi* encodes Sca proteins that mediate transport to the cell surface and are involved in several functions. The autotransporter ScaC from *O. tsutsugamushi* mediates adhesion but not the invasion of non-phagocytic mammalian cells [101]. Host cells preincubation with recombinant ScaC significantly decreased interaction with *O. tsutsugamushi*. Furthermore, a glutathione S-transferase (GST) pulldown assay confirmed fibronectin as a receptor for ScaC [101]. ScaB also mediates adhesion into epithelial and non-epithelial cells. Additionally, ScaB can mediate invasion, suggesting a role in bacterial pathogenesis [102]. Ha and colleagues demonstrated that ScaA mediates adherence in non-phagocytic host cells, including epithelial cells [103]. Interestingly, anti-ScaA antibodies diminished *O. tsutsugamushi* infection both in vitro and *in vivo*, providing protective immunity. However, these results were not observed for ScaC [103]. 

*O. tsutsugamushi* presents one of the largest known bacterial Ank repertoires. Min and colleagues have demonstrated that Ank1A, Ank1B, Ank1E, Ank1F, Ank1U4, Ank1U5, and Ank1U9 interact with Cullin1 and SKP1, two components of the SCF1 ubiquitin ligase complex that catalyzes the ubiquitination of proteins and subsequent proteasomal degradation [104]. Human elongation factor-1 alpha (EF-1α) is responsible for delivering charged amino acids to the ribosome during protein synthesis. At least six of the nine Ank proteins studied interact with EF-1α. Furthermore, Ank1U5 expression lowers EF-1α protein levels by increasing the polyubiquitination of this protein [104]. Ank4 also interacts with SKP1 and nucleates the SCF1 ubiquitin ligase complex [105]. Unfolded protein response (UPR) is a pathway that attenuates ER stress by inhibiting mRNA translation, increasing protein folding, and facilitating ER-associated degradation (ERAD). Ank4 induces UPR and inhibits ERAD. This contributes to *O. tsutsugamushi* obtaining ERAD-derived amino acids and enhancing its replication in the host cell. Finally, Ank4 binds Bat3, a chaperone important for ERAD, that is at least partially linked to ERAD modulation [105]. NF-κB is a transcription factor that is crucial in the antimicrobial response. Activation of the NF-κB pathway leads to a signaling cascade that results in the translocation of p50/p65 into the nucleus, activating antimicrobial and pro-inflammatory genes [106]. Evans and colleagues have demonstrated that Ank1 and Ank6 reduce NF-κB dependent gene expression through a complex mechanism involving interactions with p65, importin β1, and exportin 1. These two Ank factors exploit interaction with importin β1 to translocate into the nucleus and inhibit NF-κB dependent gene expression. By co-opting exportin 1, Ank1 and Ank6 also promote the decrease of p65 accumulation in the nucleus, further antagonizing NF-κB transcriptional response [106]. Furthermore, Ank1 and Ank6 interact with the SCF1 complex assembly [106]. 

Beyer and colleagues demonstrated that Ank9 perturbs the Golgi-to-ER retrograde trafficking by binding coatomer protein complex subunit [COPI] beta 2 (COPB2), which maintains Golgi stability [107]. This might contribute to bacterial intracellular replication since Golgi instability leads to increased bacterial loads inside the host cell. Additionally, Ank9 binds SKP1 and CUL1 proteins from SCF1 complex assembly, and modulates UPR and protein secretion [107]. Ank13 effector was recently identified as a bona fide nucleomodulin that interacts with the RanGDP-ankyrin repeats (RaDAR) nuclear import pathway to enter the nucleus and downregulate several host genes, including genes involved in epigenetic regulation, transcriptional control, mRNA stability, cell cycle control, and inflammatory response. Furthermore, this effector can be present in the cytoplasm modulating gene expression and reprogramming the host cell transcription by binding to SCF1 complex and transcription factors, promoting their polyubiquitination and consequent proteasomal degradation [108].

### 4.4. Rickettsia spp.

Rickettsial outer membrane protein B (rOmpB) is an autotransporter protein that is a major component of the rickettsial outer membrane [109], and for which several functions have been described. Adherence and subsequent invasion of host cells are crucial for establishing rickettsial infection [110]. rOmpB mediates the adherence and invasion of non-phagocytic cells by binding specifically to Ku70, a receptor present in the membrane of host cells [111]. rOmpB-Ku70 mediated bacterial uptake relies on actin polymerization, protein tyrosine kinase, and phosphoinositide 3 (PI3)-kinase-dependent activities and microtubule stability. Additionally, bacterial entry into the host cells is also dependent on endocytic pathway proteins c-Cbl, clathrin, and caveolin-2, suggesting their contribution to the rOmpB-Ku70 mediated internalization process [110]. Tick histone H2B is a component of the nucleosome that is also present on the surface of tick cells. Thepparit and colleagues demonstrated that rOmpB binds H2B and that the depletion of this histone results in significant inhibition of *R. felis* infection [112]. The complement system is a crucial component of innate immunity that, in response to the recognition of molecular components, becomes sequentially activated, causing a proteolytic cascade that converges with the formation of the C3 convertase that cleaves C3 [113]. This activation leads to the generation of proinflammatory mediators (anaphylatoxins), the opsonization of pathogens mediated by opsonins, and targeted lysis of the pathogen through the formation of a membrane attack complex (MAC) [114]. Riley and colleagues demonstrated that rOmpB exerts an additional function, mediating serum resistance through interaction with Factor H [109]. Factor H is a protein that acts as a regulator of the alternative pathway of the complement by disrupting C3 convertase activity. Depletion of factor H from normal human serum diminishes rickettsial survival, and factor H-binding results in lower deposition of C3 and MAC on the surface of *Rickettsia*. These results show that factor H binding partially inhibits the alternative pathway, contributing to *R. conorii* survival in serum. Finally, expression of the cleaved form of rOmpB (rOmpB β-peptide) in the outer membrane of *E. coli* is sufficient to protect the bacteria against the bactericidal properties of human serum, further supporting the rOmpB role in immune evasion [109]. Recently, Engström and colleagues demonstrated that rOmpB also promotes autophagy evasion [115]. rOmpB prevents ubiquitination of bacterial surface proteins and subsequent recognition by autophagy receptors, and is required to form a capsule-like layer. This work shows that rOmpB is essential for *R. parkeri* growth in macrophages and organ colonization in mice by acting as a protective shield to inhibit antimicrobial autophagy [115]. 

Parvulin-like peptidyl-prolylcis-transisomerase (PrsA) is an outer membrane-anchored protein that acts as a foldase which is important in folding secreted proteins, including toxins and virulence factors [116,117]. The expression of the *R. prowazekii* homolog in *E. coli* confirmed its peptidyl-prolyl cis/trans isomerase activity [118]. As observed in rOmpB, PrsA also interacts with histone H2B, facilitating infection in a tick cell line and suggesting an additional role in adhesion [112]. 

Adr2 is a rickettsial outer membrane protein that has been identified in all pathogenic species of *Rickettsia*. Garza and colleagues demonstrated that Adr2 expression in *E. coli* is sufficient to mediate serum resistance, suggesting its role in immune evasion through interaction with vitronectin, a glycoprotein that inhibits the formation of MAC, inhibiting bacterial lysis [119]. Furthermore, the role of Adr2 as an adhesin has been suggested in *R. prowazekii* and *R. rickettsii* [120]. However, the expression of Adr2 in a non-adherent strain of *E. coli* is not sufficient to mediate adherence to mammalian endothelial cells [119]. Therefore, Adr2’s role in adhesion and invasion is still conflicting and needs to be further elucidated.

Surface cell antigen 2 (Sca2), a surface protein of SFG *Rickettsia*, is another example of a protein with moonlighting activity. Cardwell and Martinez showed that expression of Sca2 in a heterologous system is sufficient to mediate adherence and invasion of mammalian epithelial and endothelial cells [121]. Additionally, it has been demonstrated that Sca2 is a bacterial actin assembly factor that functionally mimics eukaryotic formin proteins [122,123]. Sca2 nucleates unbranched actin filaments that are observed in *Rickettsia* and associates with growing barbed ends. Furthermore, this protein requires profilin (an actin-binding protein that promotes assembly) for efficient elongation [122]. Sca2 also inhibits the activity of the capping protein, promoting actin filament growth. These results demonstrate Sca2’s role in promoting actin filament nucleation and elongation to assemble actin tails, important in *Rickettsia* motility [122,123]. 

The highly conserved retropepsin-type aspartic protease APRc is also emerging as a multitasking protein. Initial work from our laboratory demonstrated that APRc catalyzes the in vitro processing of Sca5/OmpB and Sca0/OmpA autotransporter proteins that mediate adhesion and invasion of host cells by *Rickettsia*, suggesting its role as a modulator of rickettsial surface virulence factors [124]. Bacterial surface proteins that bind nonimmune immunoglobulins (Ig) are important in immune evasion because they confer protection against complement attack, decreasing opsonization and phagocytosis [125]. We have recently shown that APRc also targets host serum components, displaying nonimmune Ig-binding activity and participating in resistance to complement-mediated killing [126]. APRc binds IgG from different species (human, rabbit, and mouse) in a concentration-dependent manner and can bind different Ig classes (IgG, IgM, IgA), showing stronger interaction with IgG. Additionally, the interaction with Ig is independent of the catalytic activity. Furthermore, *E. coli* expressing the full-length protease or the catalytic mutant display resistance to complement-mediated killing, although the differences in protection (higher in the WT) suggest that APRc interferes with other serum components, whose nature we are currently exploring [126]. Unpublished data from our laboratory also anticipates a potential novel function of APRc as a modulator of cell death processes (Teixeira and Simões, unpublished), raising exciting questions on the multiple roles of this highly conserved rickettsial protease during infection.

### 4.5. Wolbachia spp. 

Although *Wolbachia* spp. do not directly infect mammalian cells, the major surface protein *Wolbachia* Surface Protein (WSP) is suggested to have an important role in mediating the host/symbiotic interactions. Brattig and colleagues demonstrated that WSP triggers the innate immune system via toll-like receptor 2 (TLR2) and TLR4 [127]. WSP increases the release of pro-inflammatory cytokines tumor necrosis factor (TNF)-α, interleukin (IL)-12, and IL-8. Interestingly, WSP also up-regulates anti-inflammatory mediators IL-10 and PGE2. These results may be related to the induction of both pro-inflammatory and anti-inflammatory molecules upon TLR stimulation, resulting in a feedback loop that maintains host homeostasis [127]. Furthermore, this outer membrane protein increases mRNA levels of IL-1β, IL-6, and tumor necrosis factor (TNF) in murine macrophages [128]. Neutrophils are crucial immune cells that control inflammation and microbial infection. WSP delays apoptosis of human neutrophils, contributing to the persistence of the inflammation [129]. Additionally, subcutaneous injection of recombinant WSP in mice induces nitric oxide producer (iNOS) mRNA expression and NOS production, important players in the immune response against bacteria that during filarial infection contribute to immune tolerance [130]. Melnikow and co-workers have later shown that WSPs were also capable of interacting with glycolytic enzymes and cytoskeleton proteins of the human filarial parasite *Brugia malayi* [131], again anticipating multiple roles for these abundant surface proteins.

## 5. Conclusions

**Figure 2:**
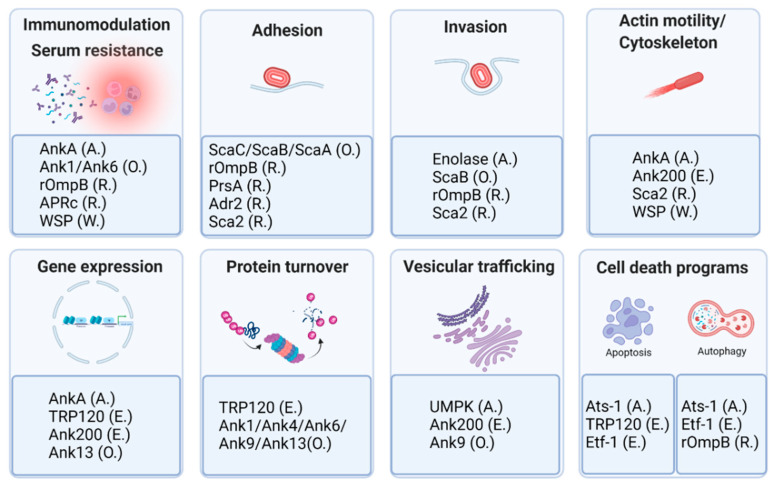
Examples of the multitasking proteins and their roles among the different members of the Rickettsiales: *Anaplasma* (A.), *Ehrlichia* (E.), *Orientia* (O.), *Rickettsia* (R.), and *Wolbachia* (W.). Details on each protein are provided in the text. Created with BioRender.com.

The examples bellow (Table 1), and summarized in Figure 2, illustrate the critical roles of many Rickettsiales’ proteins in multiple steps of the infection cycle. There are proteins like rOmpB that serve as an adhesin/invasin, help in protecting from serum bactericidal activity, and promote evasion from host antimicrobial autophagy by acting as a protective shield impairing polyubiquitination of rickettsial surface proteins. Others like *E. chaffeensis* TRP120 effector exert functions as different as ligand mimic, nucleomodulin, and E3 ubiquitin ligase (with new functions continually emerging for this and other TRP members).,or *O. tsutsugamushi* Ank effectors repertoire that are emerging as exploiters of multiple host pathways. Although the identification and characterization of multifunctional proteins in Rickettsiales is still at its nascent stage, it is clear from all the examples herein reported that these obligate organisms make extensive use of moonlight. Recruiting numerous multifunctional proteins to exert their pathogenicity enables them to extensively expand their intrinsically reduced gene repertoire. This is illustrative of the anticipated complexity when studying protein function in these obligates, as multiple independent functions may arise from differences in host cell type, intracellular localization, post-translational modifications, binding to different ligands, or oligomerization. Despite the many challenges that lie ahead to elucidate the moonlighting phenomena in these different species, we believe that the exciting data available provide an impetus for additional studies to be conducted to understand more about this emerging theme. This might not only reveal better/new therapeutic targets to treat infections caused by Rickettsiales members, but it will likely lead to the identification of yet unknown cellular mechanisms and uncover additional virulence strategies deployed by microbes.

## Figures and Tables

**Table 1:** Moonlighting proteins reported in Rickettsiales.

Organism	Protein	Activity	References
***Anaplasma phagocytophilum***	Enolase	Converts 2-phosphoglycerate to phosphoenolpyruvate in glycolysis Binds and activates plasminogen	[78,79]
	UMPK	Catalyzes the conversion of uridine monophosphate to uridine diphosphate in the cytoplasm Binds Rab10	[80]
	AnkA	Interacts with CYBB promoter and downregulates expression of CYBB and other host defense genes Binds high-content AT DNA regions and recruits HDAC1 to repress gene expression Undergoes tyrosine phosphorylation by Src kinase and interacts with neutrophil SHP-1, interfering with host cell signaling Binds adaptor protein Abi-1 and is phosphorylated by Abl-1 Interacts with actin and actin-regulating proteins α-actinin 4 and gelsolin	[83,84,85,86,87]
	Ats-1	Inhibits apoptosis in mammalian cells by inhibiting cytochrome c release and PARP cleavage Inhibits Bax-induced apoptosis in yeast cells Binds Beclin 1 and Atg14L, inducing autophagosome formation	[88,91]
	GroEL	Assists protein folding and response to stress Binds to *I. scapularis* tick cells, presenting a role in *A. phagocytophilum*-vector interaction. Interacts with HSP70 and MSP4	[92]
	HSP70	Assists protein folding and response to stress Binds to *I. scapularis* tick cells, presenting a role in *A. phagocytophilum*-vector interaction. Interacts with GroEL and MSP4	[92]
***Ehrlichia chaffeensis***	TRP120	Interacts with Wnt and Notch receptors to activate both pathways, regulating apoptosis and autophagy Modulates the expression of several genes Interacts with GC-rich regions of DNA Targets host proteins to degradation, regulating signaling pathways	[93,95,96,97]
	Ank200	Targets transcriptional factors, genes of the epigenetic machinery and components of signaling pathways Interacts with genes related to vesicular trafficking and cytoskeleton proteins Potentially contributes to the delivery of nutrients to ehrlichial inclusions	[94]
	Etf-1	Inhibits apoptosis in mammalian cells and yeast Increases mitochondrial MnSod Decreases ROS, preventing cellular damage and apoptosis Modulates autophagy by recruiting GTP-bound RAB5 and binding VPS34 and Beclin1	[98,100]
***Orientia tsutsugamushi***	ScaC	Mediates transport to the cell surface Mediates adhesion in non-phagocytic mammalian cells Binds fibronectin	[101]
	ScaB	Mediates transport to the cell surface Mediates adhesion and invasion into epithelial and non-epithelial cells	[102]
	ScaA	Mediates transport to the cell surface Mediates adhesion in non-phagocytic mammalian cells	[103]
	Ank1A	Interacts with SKP1 Interacts with EF-1α	[104]
	Ank1B	Interacts with Cullin1 and SKP1 Interacts with EF-1α	[104]
	Ank1E	Interacts with Cullin1 and SKP1 Interacts with EF-1α	[104]
	Ank1U4	Interacts with Cullin1 and SKP1 Interacts with EF-1α	[104]
	Ank1U5	Interacts with Cullin1 and SKP1 Decreases EF-1α protein level	[104]
	Ank1U9	Interacts with Cullin1 and SKP1 Interacts with EF-1α	[104]
	Ank4	Interacts with SKP1 Nucleates SCF1 ubiquitin ligase complex Induces UPR and inhibits ERAD, contributing bacterial obtaining of ERAD-derived amino acids Binds Bat3	[105]
	Ank1	Decreases NF-κB dependent gene expression by interacting with importin β1, p65 and exportin Interacts with SCF1 complex assembly	[106]
	Ank6	Decreases NF-κB dependent gene expression by interacting with importin β1, p65 and exportin Interacts with SCF1 complex assembly	[106]
	Ank9	Perturbs Golgi-to-ER retrograde trafficking by binding COPB2 Binds SKP1 and CUL1 proteins, modulating UPR Binds SCF1 complex and transcription factors, modulating protein secretion	[107]
	Ank13	Interacts with RaDAR and downregulates multiple host genes, acting as a nucleomodulin Modulates cytoplasm gene expression Reprograms host cell transcription	[108]
***Rickettsia* spp.**	rOmpB	Autotransporter Mediates adherence and invasion of non-phagocytic cells by binding Ku70 Binds H2B and facilitates infection in a tick cell line Mediates serum resistance by interacting with Factor H Promotes autophagy evasion (Prevents ubiquitination of bacterial surface proteins and subsequent recognition by autophagy receptors)	[109,110,111,112,115]
	PrsA	Mediates folding of secreted proteins Interacts with H2B and facilitates infection in a tick cell line	[112,116,117,118]
	Adr2	Interacts with vibronectin and mediates serum resistance May act as an adhesin	[119,120]
	Sca2	Mediates adherence and invasion of mammalian epithelial and endothelial cells Promotes actin filament nucleation and elongation to assemble actin tails Inhibits capping proteins’ activity	[121,122,123]
	APRc	Catalyzes in vitro processing of Sca5/OmpB and Sca0/OmpA Presents nonimmune Ig-binding activity & Targets host serum components Confers resistance to complement-mediated killing	[124,126]
***Wolbachia* spp.**	WSP	Triggers innate immune system by interacting with TLR2 and TLR4 Delays apoptosis of human neutrophils Induces iNOS mRNA expression and NOS production Interacts with glycolytic enzymes and cytoskeleton proteins of *Brugia malayi*	[127,128,129,130,131]

**Abbreviations**: Abi-1: Abl-interactor 1, Ank200: 200 kDa ankyrin repeat protein, AnkA: ankyrin repeat domain-containing protein A, APRc: aspartic protease from *Rickettsia conorii*, Ats-1: *Anaplasma* translocated factor 1, COPB2: coatomer protein complex subunit beta 2, EF-1α: elongation factor-1 alpha, ER: endoplasmic reticulum, ERAD: ER-associated degradation, Etf-1: ehrlichial translocated factor 1, HDAC1: histone deacetylase 1, HSP70: 70-kDa heat shock protein, Ig: immunoglobulins, IL-8: interleukin 8, iNOS: nitric oxide producer, MnSod: manganese superoxide dismutase, MSP4: major surface protein 4, PrsA: parvulin-like peptidyl-prolylcis-transisomerase, RaDAR: RanGDP-ankyrin repeats, rOmpB: rickettsial outer membrane protein B, ROS: reactive oxygen species, Sca2: surface cell antigen 2, SHP-1: Src homology phosphatase-1, TGN: trans Golgi network, TLR2: Toll-like receptor 2, TNF-α: tumor necrosis factor alpha, TRP: tandem repeat protein, UMPK: uridine monophosphate kinase, UPR. unfolded protein response, WSP: *Wolbachia* surface protein.

## References

[1] Salje J. (2021). Cells within cells: Rickettsiales and the obligate intracellular bacterial lifestyle. Nat. Rev. Genet..

[2] Khatat S.E.H., Daminet S., Duchateau L., Elhachimi L., Kachani M., Sahibi H. (2021). Epidemiological and Clinicopathological Features of *Anaplasma phagocytophilum* Infection in Dogs: A Systematic Review. Front. Veter.-Sci..

[3] Matei I.A., Estrada-Peña A., Cutler S.J., Vayssier-Taussat M., Castro L.V., Potkonjak A., Zeller H., Mihalca A.D. (2019). A review on the eco-epidemiology and clinical management of human granulocytic anaplasmosis and its agent in Europe. Parasites Vectors.

[4] Zhang L., Cui F., Wang L., Zhang L., Zhang J., Wang S., Yang S. (2011). Investigation of anaplasmosis in Yiyuan County, Shandong Province, China. Asian Pac. J. Trop. Med..

[5] Gettings J.R., Self S.C.W., McMahan C.S., Brown D.A., Nordone S.K., Yabsley M.J. (2020). Local and regional temporal trends (2013–2019) of canine *Ehrlichia* spp. seroprevalence in the USA. Parasites Vectors.

[6] Luce-Fedrow A., Lehman M.L., Kelly D.J., Mullins K., Maina A.N., Stewart R.L., Ge H., John H.S., Jiang J., Richards A.L. (2018). A Review of Scrub Typhus (*Orientia tsutsugamushi* and Related Organisms): Then, Now, and Tomorrow. Trop. Med. Infect. Dis..

[7] Chakraborty S., Sarma N. (2017). Scrub Typhus: An Emerging Threat. Indian J. Dermatol..

[8] Parola P., Paddock C.D., Socolovschi C., Labruna M.B., Mediannikov O., Kernif T., Abdad M.Y., Stenos J., Bitam I., Fournier P.-E. (2013). Update on Tick-Borne Rickettsioses around the World: A Geographic Approach. Clin. Microbiol. Rev..

[9] European Centre for Disease Prevention and Control (2013). Epidemiological Situation of Rickettsioses in EU/EFTA Countries.

[10] Binder A.M., Armstrong P.A. (2019). Increase in Reports of Tick-Borne Rickettsial Diseases in the United States. Am. J. Nurs..

[11] Drexler N.A., Dahlgren F., Massung R.F., Behravesh C.B., Heitman K.N., Paddock C.D. (2016). National Surveillance of Spotted Fever Group Rickettsioses in the United States, 2008–2012. Am. J. Trop. Med. Hyg..

[12] Álvarez-Hernández G., Roldán J.F.G., Milan N.S.H., Lash R.R., Behravesh C.B., Paddock C.D. (2017). Rocky Mountain spotted fever in Mexico: Past, present, and future. Lancet Infect. Dis..

[13] Spernovasilis N., Markaki I., Papadakis M., Mazonakis N., Ierodiakonou D. (2021). Mediterranean Spotted Fever: Current Knowledge and Recent Advances. Trop. Med. Infect. Dis..

[14] Buczek W., Koman-Iżko A., Buczek A.M., Bartosik K., Kulina D., Ciura D. (2020). Spotted fever group rickettsiae transmitted by Dermacentor ticks and determinants of their spread in Europe. Ann. Agric. Environ. Med..

[15] Heinrich N., Dill T., Dobler G., Clowes P., Kroidl I., Starke M., Ntinginya N.E., Maboko L., Löscher T., Hoelscher M. (2015). High Seroprevalence for Spotted Fever Group Rickettsiae, Is Associated with Higher Temperatures and Rural Environment in Mbeya Region, Southwestern Tanzania. PLoS Negl. Trop. Dis..

[16] Martinez M.A.C., Ramírez-Hernández A., Blanton L.S. (2021). Manifestations and Management of Flea-Borne Rickettsioses. Res. Rep. Trop. Med..

[17] Brown L.D., Macaluso K.R. (2016). Rickettsia felis, an Emerging Flea-Borne Rickettsiosis. Curr. Trop. Med. Rep..

[18] Maina A.N., Jiang J., Luce-Fedrow A., John H.K.S., Farris C.M., Richards A.L. (2019). Worldwide Presence and Features of Flea-Borne Rickettsia asembonensis. Front. Veter.-Sci..

[19] Piotrowski M., Rymaszewska A. (2020). Expansion of Tick-Borne Rickettsioses in the World. Microorganisms.

[20] Jeffery C.J. (1999). Moonlighting proteins. Trends Biochem. Sci..

[21] Chen C., Liu H., Zabad S., Rivera N., Rowin E., Hassan M., De Jesus S.M.G., Santos P.S.L., Kravchenko K., Mikhova M. (2020). MoonProt 3.0: An update of the moonlighting proteins database. Nucleic Acids Res..

[22] Franco-Serrano L., Hernández S., Calvo A., Severi M.A., Ferragut G., Pérez-Pons J., Piñol J., Pich Ò., Mozo-Villarias Á., Amela I. (2018). MultitaskProtDB-II: An update of a database of multitasking/moonlighting proteins. Nucleic Acids Res..

[23] Huberts D.H., van der Klei I.J. (2010). Moonlighting proteins: An intriguing mode of multitasking. Biochim. Biophys. Acta.

[24] Jeffery C.J. (2015). Why study moonlighting proteins?. Front. Genet..

[25] Hernandez S., Franco L., Calvo A., Ferragut G., Hermoso A., Amela I., Gomez A., Querol E., Cedano J. (2015). Bioinformatics and Moonlighting Proteins. Front. Bioeng. Biotechnol..

[26] Khan I.K., Bhuiyan M., Kihara D. (2017). DextMP: Deep dive into text for predicting moonlighting proteins. Bioinformatics.

[27] Shirafkan F., Gharaghani S., Rahimian K., Sajedi R.H., Zahiri J. (2021). Moonlighting protein prediction using physico-chemical and evolutional properties via machine learning methods. BMC Bioinform..

[28] Ribeiro D.M., Briere G., Bely B., Spinelli L., Brun C. (2018). MoonDB 2.0: An updated database of extreme multifunctional and moonlighting proteins. Nucleic Acids Res..

[29] Jeffery C.J. (2017). Protein moonlighting: What is it, and why is it important?. Philos. Trans. R. Soc. B Biol. Sci..

[30] Wang G., Xia Y., Cui J., Gu Z., Song Y., Chen Y.Q., Chen H., Zhang H., Chen W. (2014). The Roles of Moonlighting Proteins in Bacteria. Curr. Issues Mol. Biol..

[31] Espinosa-Cantú A., Cruz-Bonilla E., Noda-Garcia L., DeLuna A. (2020). Multiple Forms of Multifunctional Proteins in Health and Disease. Front. Cell Dev. Biol..

[32] Jeffery C. (2018). Intracellular proteins moonlighting as bacterial adhesion factors. AIMS Microbiol..

[33] Sharma A.K., Dhasmana N., Dubey N., Kumar N., Gangwal A., Gupta M., Singh Y. (2017). Bacterial Virulence Factors: Secreted for Survival. Indian J. Microbiol..

[34] Henderson B., Martin A. (2011). Bacterial Virulence in the Moonlight: Multitasking Bacterial Moonlighting Proteins Are Virulence Determinants in Infectious Disease. Infect. Immun..

[35] Kainulainen V., Korhonen T.K. (2014). Dancing to Another Tune—Adhesive Moonlighting Proteins in Bacteria. Biology.

[36] Pancholi V., Fischetti V.A. (1992). A major surface protein on group A streptococci is a glyceraldehyde-3-phosphate-dehydrogenase with multiple binding activity. J. Exp. Med..

[37] Amblee V., Jeffery C.J. (2015). Physical Features of Intracellular Proteins that Moonlight on the Cell Surface. PLoS ONE.

[38] Dallo S.F., Kannan T.R., Blaylock M.W., Baseman J.B. (2002). Elongation factor Tu and E1 β subunit of pyruvate dehydrogenase complex act as fibronectin binding proteins in Mycoplasma pneumoniae. Mol. Microbiol..

[39] Kunert A., Losse J., Gruszin C., Hühn M., Kaendler K., Mikkat S., Volke D., Hoffmann R., Jokiranta T.S., Seeberger H. (2007). Immune Evasion of the Human Pathogen *Pseudomonas aeruginosa*: Elongation Factor Tuf is a Factor H and Plasminogen Binding Protein. J. Immunol..

[40] Culp E., Wright G.D. (2017). Bacterial proteases, untapped antimicrobial drug targets. J. Antibiot..

[41] Lebrun I., Marques-Porto R., Pereira A., Perpetuo E. (2009). Bacterial Toxins: An Overview on Bacterial Proteases and their Action as Virulence Factors. Mini-Rev. Med. Chem..

[42] Rogers L.D., Overall C.M. (2013). Proteolytic Post-translational Modification of Proteins: Proteomic Tools and Methodology. Mol. Cell. Proteom..

[43] Beckmann C., Waggoner J.D., Harris T.O., Tamura G.S., Rubens C.E. (2002). Identification of Novel Adhesins from Group B Streptococci by Use of Phage Display Reveals that C5a Peptidase Mediates Fibronectin Binding. Infect. Immun..

[44] Torres A., Chamorro-Veloso N., Costa P., Cádiz L., Del Canto F., Venegas S., Nitsche M.L., Coloma-Rivero R., Montero D., Vidal R. (2020). Deciphering Additional Roles for the EF-Tu, l-Asparaginase II and OmpT Proteins of Shiga Toxin-Producing *Escherichia coli*. Microorganisms.

[45] Wan L., Guo Y., Hui C.-Y., Liu X.-L., Zhang W.-B., Cao H. (2014). The surface protease ompT serves as Escherichia coli K1 adhesin in binding to human brain micro vascular endothelial cells. Pak. J. Pharm. Sci..

[46] Leon-Sicairos N., Reyes-Cortes R., Guadron-Llanos A.M., Madueña-Molina J., Leon-Sicairos C., Canizalez-Roman A. (2015). Strategies of Intracellular Pathogens for Obtaining Iron from the Environment. BioMed Res. Int..

[47] Bourdonnay E., Henry T. (2016). Catch me if you can. eLife.

[48] Mak T.W., Saunders M.E., Mak T.W., Saunders M.E. (2006). 22-Immunity to Pathogens. The Immune Response.

[49] Eisenreich W., Rudel T., Heesemann J., Goebel W. (2019). How Viral and Intracellular Bacterial Pathogens Reprogram the Metabolism of Host Cells to Allow Their Intracellular Replication. Front. Cell. Infect. Microbiol..

[50] Köseoğlu V.K., Agaisse H. (2019). Evolutionary Perspectives on the Moonlighting Functions of Bacterial Factors That Support Actin-Based Motility. mBio.

[51] Ensgraber M., Loos M. (1992). A 66-kilodalton heat shock protein of Salmonella typhimurium is responsible for binding of the bacterium to intestinal mucus. Infect. Immun..

[52] Jagadeesan B., Koo O.K., Kim K.-P., Burkholder K.M., Mishra K.K., Aroonnual A., Bhunia A.K. (2010). LAP, an alcohol acetaldehyde dehydrogenase enzyme in Listeria, promotes bacterial adhesion to enterocyte-like Caco-2 cells only in pathogenic species. Microbiology.

[53] Wampler J.L., Kim K.-P., Jaradat Z., Bhunia A.K. (2004). Heat Shock Protein 60 Acts as a Receptor for the Listeria Adhesion Protein in Caco-2 Cells. Infect. Immun..

[54] Garduño R.A., Garduño E., Hoffman P.S. (1998). Surface-Associated Hsp60 Chaperonin of Legionella pneumophila Mediates Invasion in a HeLa Cell Model. Infect. Immun..

[55] Sharma D., Masison D.C. (2009). Hsp70 Structure, Function, Regulation and Influence on Yeast Prions. Protein Pept. Lett..

[56] Liu H., Jeffery C.J. (2020). Moonlighting Proteins in the Fuzzy Logic of Cellular Metabolism. Molecules.

[57] Kinhikar A.G., Vargas D., Li H., Mahaffey S.B., Hinds L., Belisle J.T., Laal S. (2006). Mycobacterium tuberculosis malate synthase is a laminin-binding adhesin. Mol. Microbiol..

[58] Xolalpa W., Vallecillo A.J., Lara M., Mendoza-Hernandez G., Comini M., Spallek R., Singh M., Espitia C. (2007). Identification of novel bacterial plasminogen-binding proteins in the human pathogen *Mycobacterium tuberculosis*. Proteomics.

[59] Heilmann C., Thumm G., Chhatwal G.S., Hartleib J., Uekötter A., Peters G. (2003). Identification and characterization of a novel autolysin (Aae) with adhesive properties from *Staphylococcus epidermidis*. Microbiology.

[60] Heilmann C., Hartleib J., Hussain M.S., Peters G. (2005). The Multifunctional Staphylococcus aureus Autolysin Aaa Mediates Adherence to Immobilized Fibrinogen and Fibronectin. Infect. Immun..

[61] Milohanic E., Pron B., Berche P., Gaillard J.-L., The European Listeria Genome Consortium (2000). Identification of new loci involved in adhesion of Listeria monocytogenes to eukaryotic cells The GenBank accession numbers for the sequences determined in this work are AF104224–AF104229. Microbiology.

[62] Dumler J.S., Walker D.H., Garrity G.M., Brenner D.J., Krieg N.R., Staley J.T. (2005). Order II. Rickettsiales. Bergey’s Manual of Systematic Bacteriology.

[63] Vescovo I.A.L., Golemba M.D., Di Lello F.A., Culasso A.C., Levin G., Ruberto L., Mac Cormack W.P., López J.L. (2014). Rich bacterial assemblages from Maritime Antarctica (Potter Cove, South Shetlands) reveal several kinds of endemic and undescribed phylotypes. Rev. Argent. Microbiol..

[64] McQuiston J.H., Paddock C.D. (2014). Public Health: Rickettsial Infections and Epidemiology. Intracellular Pathogens II.

[65] Wood H., Artsob H. (2012). Spotted Fever Group Rickettsiae: A Brief Review and a Canadian Perspective. Zoonoses Public Health.

[66] Dumler J.S. (2014). Clinical Disease: Current Treatment and New Challenges. Intracellular Pathogens II.

[67] Sahni A., Fang R., Sahni S.K., Walker D.H. (2019). Pathogenesis of Rickettsial Diseases: Pathogenic and Immune Mechanisms of an Endotheliotropic Infection. Annu. Rev. Pathol. Mech. Dis..

[68] Kelly D.J., Richards A.L., Temenak J., Strickman D., Dasch G.A. (2002). The Past and Present Threat of Rickettsial Diseases to Military Medicine and International Public Health. Clin. Infect. Dis..

[69] Xu G., Walker D.H., Jupiter D., Melby P.C., Arcari C.M. (2017). A review of the global epidemiology of scrub typhus. PLoS Negl. Trop. Dis..

[70] Devasagayam E., Dayanand D., Kundu D., Kamath M.S., Kirubakaran R., Varghese G.M. (2021). The burden of scrub typhus in India: A systematic review. PLoS Negl. Trop. Dis..

[71] El-Sayed A., Kamel M. (2020). Climatic changes and their role in emergence and re-emergence of diseases. Environ. Sci. Pollut. Res..

[72] Wu T., Perrings C., Kinzig A., Collins J.P., Minteer B.A., Daszak P. (2017). Economic growth, urbanization, globalization, and the risks of emerging infectious diseases in China: A review. Ambio.

[73] Blazejak K., Janecek E., Strube C. (2017). A 10-year surveillance of Rickettsiales (*Rickettsia* spp. and *Anaplasma phagocytophilum*) in the city of Hanover, Germany, reveals Rickettsia spp. as emerging pathogens in ticks. Parasites Vectors.

[74] John R., Varghese G.M. (2020). Scrub typhus: A reemerging infection. Curr. Opin. Infect. Dis..

[75] Alkishe A., Raghavan R.K., Peterson A.T. (2021). Likely Geographic Distributional Shifts among Medically Important Tick Species and Tick-Associated Diseases under Climate Change in North America: A Review. Insects.

[76] Nelder M.P., Russell C.B., Lindsay L.R., Dibernardo A., Brandon N.C., Pritchard J., Johnson S., Cronin K., Patel S.N. (2019). Recent Emergence of *Anaplasma phagocytophilum* in Ontario, Canada: Early Serological and Entomological Indicators. Am. J. Trop. Med. Hyg..

[77] Li T., Yang Z., Dong Z., Wang M. (2014). Meteorological factors and risk of scrub typhus in Guangzhou, southern China, 2006–2012. BMC Infect. Dis..

[78] Rikihisa Y. (2011). Mechanisms of Obligatory Intracellular Infection with *Anaplasma phagocytophilum*. Clin. Microbiol. Rev..

[79] Gao X., Zheng C., Liu X. (2018). Expression, purification, and biological characterization of *Anaplasma phagocytophilum* enolase. Biosci. Trends.

[80] Truchan H.K., VieBrock L., Cockburn C.L., Ojogun N., Griffin B.P., Wijesinghe D.S., Chalfant C.E., Carlyon J.A. (2016). *Anaplasma phagocytophilum* Rab10-dependent parasitism of thetrans-Golgi network is critical for completion of the infection cycle. Cell. Microbiol..

[81] English A.R., Voeltz G.K. (2013). Rab10 GTPase regulates ER dynamics and morphology. Nat. Cell Biol..

[82] Park J., Kim K.J., Choi K.-S., Grab D.J., Dumler J.S. (2004). *Anaplasma phagocytophilum* AnkA binds to granulocyte DNA and nuclear proteins. Cell. Microbiol..

[83] Garcia-Garcia J.C., Rennoll-Bankert K.E., Pelly S., Milstone A.M., Dumler J.S. (2009). Silencing of Host Cell CYBB Gene Expression by the Nuclear Effector AnkA of the Intracellular Pathogen *Anaplasma phagocytophilum*. Infect. Immun..

[84] Rennoll-Bankert K. (2012). Lessons from *Anaplasma phagocytophilum*: Chromatin Remodeling by Bacterial Effectors. Infect. Disord.-Drug Targets.

[85] Ijdo J.W., Carlson A.C., Kennedy E.L. (2007). *Anaplasma phagocytophilum* AnkA is tyrosine-phosphorylated at EPIYA motifs and recruits SHP-1 during early infection. Cell. Microbiol..

[86] Lin M., Dulk-Ras A.D., Hooykaas P.J.J., Rikihisa Y. (2007). *Anaplasma phagocytophilum* AnkA secreted by type IV secretion system is tyrosine phosphorylated by Abl-1 to facilitate infection. Cell. Microbiol..

[87] Rikihisa Y. (2017). Role and Function of the Type IV Secretion System in Anaplasma and Ehrlichia Species. Type IV Secretion in Gram-Negative and Gram-Positive Bacteria.

[88] Niu H., Kozjak-Pavlovic V., Rudel T., Rikihisa Y. (2010). *Anaplasma phagocytophilum* Ats-1 Is Imported into Host Cell Mitochondria and Interferes with Apoptosis Induction. PLoS Pathog..

[89] Friedrich A., Pechstein J., Berens C., Lührmann A. (2017). Modulation of host cell apoptotic pathways by intracellular pathogens. Curr. Opin. Microbiol..

[90] Niu H., Rikihisa Y. (2013). Ats-1: A novel bacterial molecule that links autophagy to bacterial nutrition. Autophagy.

[91] Niu H., Xiong Q., Yamamoto A., Hayashi-Nishino M., Rikihisa Y. (2012). Autophagosomes induced by a bacterial Beclin 1 binding protein facilitate obligatory intracellular infection. Proc. Natl. Acad. Sci. USA.

[92] Villar M., Ayllón N., Kocan K.M., Bonzón-Kulichenko E., Alberdi P., Blouin E.F., Weisheit S., Mateos-Hernández L., Cabezas-Cruz A., Bell-Sakyi L. (2015). Identification and Characterization of *Anaplasma phagocytophilum* Proteins Involved in Infection of the Tick Vector, Ixodes scapularis. PLoS ONE.

[93] Byerly C.D., Patterson L.L., McBride J.W. (2021). Ehrlichia TRP effectors: Moonlighting, mimicry and infection. Pathog. Dis..

[94] Dunphy P.S., Luo T., McBride J.W. (2013). Ehrlichia moonlighting effectors and interkingdom interactions with the mononuclear phagocyte. Microbes Infect..

[95] Kumagai Y., Matsuo J., Hayakawa Y., Rikihisa Y. (2010). Cyclic di-GMP Signaling Regulates Invasion by Ehrlichia chaffeensis of Human Monocytes. J. Bacteriol..

[96] Rogan M.R., Patterson L.L., Byerly C.D., Luo T., Paessler S., Veljkovic V., Quade B., McBride J.W. (2021). Ehrlichia chaffeensis TRP120 Is a Wnt Ligand Mimetic That Interacts with Wnt Receptors and Contains a Novel Repetitive Short Linear Motif That Activates Wnt Signaling. mSphere.

[97] Klema V.J., Sepuru K.M., Füllbrunn N., Farris T.R., Dunphy P.S., McBride J.W., Rajarathnam K., Choi K.H. (2018). Ehrlichia chaffeensis TRP120 nucleomodulin binds DNA with disordered tandem repeat domain. PLoS ONE.

[98] Liu H., Bao W., Lin M., Niu H., Rikihisa Y. (2012). Ehrlichia type IV secretion effector ECH0825 is translocated to mitochondria and curbs ROS and apoptosis by upregulating host MnSOD. Cell. Microbiol..

[99] Backert S., Grohmann E. (2018). Type IV Secretion in Gram-Negative and Gram-Positive Bacteria.

[100] Lin M., Liu H., Xiong Q., Niu H., Cheng Z., Yamamoto A., Rikihisa Y. (2016). Ehrlichia secretes Etf-1 to induce autophagy and capture nutrients for its growth through RAB5 and class III phosphatidylinositol 3-kinase. Autophagy.

[101] Ha N.-Y., Cho N.-H., Kim Y.-S., Choi M.-S., Kim I.-S. (2011). An Autotransporter Protein from *Orientia tsutsugamushi* Mediates Adherence to Nonphagocytic Host Cells. Infect. Immun..

[102] Nguyen Y.T.H., Kim C., Kim Y., Jeon K., Kim H.-I., Ha N.-Y., Cho N.-H. (2021). The *Orientia tsutsugamushi* ScaB Autotransporter Protein Is Required for Adhesion and Invasion of Mammalian Cells. Front. Microbiol..

[103] Ha N.-Y., Sharma P., Kim G., Kim Y., Min C.-K., Choi M.-S., Kim I.-S., Cho N.-H. (2015). Immunization with an Autotransporter Protein of *Orientia tsutsugamushi* Provides Protective Immunity against Scrub Typhus. PLoS Negl. Trop. Dis..

[104] Min C.-K., Kwon Y.-J., Ha N.-Y., Cho B.-A., Kim J.-M., Kwon E.-K., Kim Y.-S., Choi M.-S., Kim I.-S., Cho N.-H. (2014). Multiple *Orientia tsutsugamushi* Ankyrin Repeat Proteins Interact with SCF1 Ubiquitin Ligase Complex and Eukaryotic Elongation Factor 1 α. PLoS ONE.

[105] Rodino K.G., VieBrock L., Evans S.M., Ge H., Richards A.L., Carlyon J.A. (2018). *Orientia tsutsugamushi* Modulates Endoplasmic Reticulum-Associated Degradation To Benefit Its Growth. Infect. Immun..

[106] Evans S.M., Rodino K.G., Adcox H.E., Carlyon J.A. (2018). *Orientia tsutsugamushi* uses two Ank effectors to modulate NF-κB p65 nuclear transport and inhibit NF-κB transcriptional activation. PLoS Pathog..

[107] Beyer A.R., Rodino K.G., VieBrock L., Green R.S., Tegels B.K., Oliver L.D., Marconi R.T., Carlyon J.A. (2017). *Orientia tsutsugamushi* Ank9 is a multifunctional effector that utilizes a novel GRIP-like Golgi localization domain for Golgi-to-endoplasmic reticulum trafficking and interacts with host COPB. Cell Microbiol..

[108] Adcox H.E., Hatke A.L., Andersen S.E., Gupta S., Otto N.B., Weber M.M., Marconi R.T., Carlyon J.A. (2021). *Orientia tsutsugamushi* Nucleomodulin Ank13 Exploits the RaDAR Nuclear Import Pathway To Modulate Host Cell Transcription. mBio.

[109] Riley S.P., Patterson J.L., Martinez J.J. (2012). The Rickettsial OmpB β-Peptide of Rickettsia conorii Is Sufficient To Facilitate Factor H-Mediated Serum Resistance. Infect. Immun..

[110] Chan Y.G.Y., Cardwell M.M., Hermanas T.M., Uchiyama T., Martinez J.J. (2009). Rickettsial outer-membrane protein B (rOmpB) mediates bacterial invasion through Ku70 in an actin, c-Cbl, clathrin and caveolin 2-dependent manner. Cell. Microbiol..

[111] Walker D.H., Ismail N. (2008). Emerging and re-emerging rickettsioses: Endothelial cell infection and early disease events. Nat. Rev. Genet..

[112] Thepparit C., Bourchookarn A., Petchampai N., Barker S.A., Macaluso K.R. (2010). Interaction of Rickettsia felis with histone H2B facilitates the infection of a tick cell line. Microbiology.

[113] Merle N.S., Noé R., Halbwachs-Mecarelli L., Fremeaux-Bacchi V., Roumenina L.T. (2015). Complement System Part II: Role in Immunity. Front. Immunol..

[114] Dunkelberger J.R., Song W.-C. (2010). Complement and its role in innate and adaptive immune responses. Cell Res..

[115] Engström P., Burke T., Mitchell G., Ingabire N., Mark K.G., Golovkine G., Iavarone A.T., Rape M., Cox J.S., Welch M.D. (2019). Evasion of autophagy mediated by Rickettsia surface protein OmpB is critical for virulence. Nat. Microbiol..

[116] Emelyanov V.V., Demyanova N.G. (1999). Nucleotide sequence of the gene and features of the major outer membrane protein of a virulent Rickettsia prowazekii strain. Biochemistry.

[117] Jakob R.P., Koch J.R., Burmann B., Schmidpeter P.A., Hunkeler M., Hiller S., Schmid F.X., Maier T. (2015). Dimeric Structure of the Bacterial Extracellular Foldase PrsA. J. Biol. Chem..

[118] Loukianov E.V., Emelyanov V.V. (2004). A 29.5 KDA Heat-modifiable Major Outer Membrane Protein of Rickettsia Prowazekii, Putative Virulence Factor, is a Peptidyl-Prolyl Cis / trans Isomerase. IUBMB Life.

[119] Garza D.A., Riley S.P., Martinez J.J. (2017). Expression of Rickettsia Adr2 protein in E. coli is sufficient to promote resistance to complement-mediated killing, but not adherence to mammalian cells. PLoS ONE.

[120] Vellaiswamy M., Kowalczewska M., Merhej V., Nappez C., Vincentelli R., Renesto P., Raoult D. (2011). Characterization of rickettsial adhesin Adr2 belonging to a new group of adhesins in α-proteobacteria. Microb. Pathog..

[121] Cardwell M.M., Martinez J.J. (2009). The Sca2 Autotransporter Protein from Rickettsia conorii Is Sufficient To Mediate Adherence to and Invasion of Cultured Mammalian Cells. Infect. Immun..

[122] Haglund C.M., Choe J.E., Skau C.T., Kovar D.R., Welch M.D. (2010). Rickettsia Sca2 is a bacterial formin-like mediator of actin-based motility. Nat. Cell Biol..

[123] Madasu Y., Suarez C., Kast D., Kovar D.R., Dominguez R. (2013). Rickettsia Sca2 has evolved formin-like activity through a different molecular mechanism. Proc. Natl. Acad. Sci. USA.

[124] Cruz R., Huesgen P., Riley S.P., Wlodawer A., Faro C., Overall C.M., Martinez J.J., Simões I. (2014). RC1339/APRc from Rickettsia conorii Is a Novel Aspartic Protease with Properties of Retropepsin-Like Enzymes. PLoS Pathog..

[125] Sidorin E.V., Solov’Eva T.F. (2011). IgG-binding proteins of bacteria. Biochemistry.

[126] Curto P., Barro A., Almeida C., Vieira-Pires R.S., Simões I. (2021). The Retropepsin-Type Protease APRc as a Novel Ig-Binding Protein and Moonlighting Immune Evasion Factor of Rickettsia. mBio.

[127] Brattig N.W., Bazzocchi C., Kirschning C.J., Reiling N., Büttner D.W., Ceciliani F., Geisinger F., Hochrein H., Ernst M., Wagner H. (2004). The Major Surface Protein of Wolbachia Endosymbionts in Filarial Nematodes Elicits Immune Responses through TLR2 and TLR4. J. Immunol..

[128] Porksakorn C., Nuchprayoon S., Park K., Scott A.L. (2007). Proinflammatory Cytokine Gene Expression by Murine Macrophages in Response toBrugia malayi WolbachiaSurface Protein. Mediat. Inflamm..

[129] Bazzocchi C., Comazzi S., Santoni R., Bandi C., Genchi C., Mortarino M. (2007). Wolbachia surface protein (WSP) inhibits apoptosis in human neutrophils. Parasite Immunol..

[130] Morchón R., Bazzocchi C., López-Belmonte J., Martín-Pacho J.R., Kramer L.H., Grandi G., Simón F. (2007). iNOs expression is stimulated by the major surface protein (rWSP) from Wolbachia bacterial endosymbiont of Dirofilaria immitis following subcutaneous injection in mice. Parasitol. Int..

[131] Melnikow E., Xu S., Liu J., Bell A.J., Ghedin E., Unnasch T.R., Lustigman S. (2013). A Potential Role for the Interaction of Wolbachia Surface Proteins with the Brugia malayi Glycolytic Enzymes and Cytoskeleton in Maintenance of Endosymbiosis. PLoS Negl. Trop. Dis..

